# From genome assembly to functional interpretation: an epistemological shift in understanding plant mitochondrial architecture

**DOI:** 10.3389/fpls.2025.1723739

**Published:** 2025-12-15

**Authors:** Tianchao Wang, Hao He, Jinwei Sun, Jie Man, Jiawei Li, Chaohua Sun, Yanjie Zhao, Zheng Kuang, Yifei Liu, Zhenyu Huang, Yongxin Zhao

**Affiliations:** 1 Institute of Biotechnology, Beijing Academy of Agriculture and Forestry Sciences, Beijing, China; 2 Houji Zhilian Information Technology Co., Ltd, Beijing, China; 3 Tongzhou International Seed Industry Technology Co., Ltd, Beijing, China; 4 College of Horticulture and Forestry, Tarim University, Aral, China; 5 Zhengzhou Fruit Research Institute, Chinese Academy of Agricultural Sciences, Zhengzhou, China

**Keywords:** plant mitochondrial genome, genome assembly, long-read assembly, graph-based assembly, algorithm evaluation

## Abstract

The plant mitochondrial genome exhibits high complexity and structural diversity, which pose major challenges to its accurate assembly and in-depth interpretation. In recent years, the rapid advancement of sequencing technologies has driven the development of multiple assembly tools tailored for plant mitochondrial genomes. This review systematically summarizes recent progress in plant mitochondrial genome assembly, focusing on several representative computational tools, including GetOrganelle, GSAT, HIMT, PMAT, TIPPo, and PMAT2, and discusses their algorithmic principles, major advantages, and potential limitations. Furthermore, we highlight the methodological transition of mitochondrial genomics from traditional static sequence reconstruction to dynamic structural modeling. Finally, we outline future perspectives in data integration, standardization, and community benchmarking, which will collectively contribute to establishing a unified analytical framework for interpreting the complexity of plant mitochondrial genomes.

## 1 Introduction

Plant mitochondria serve as the central hub of energy metabolism in plant cells. The plant mitochondrial genome (mitogenome), which resides within the mitochondria, contains genetic information encoding key proteins of the respiratory chain complexes, as well as rRNAs, tRNAs, and genes involved in energy metabolism and the maintenance of mitochondrial integrity (Kubo and Newton, 2008; Gualberto and Newton, 2017). Both mitochondria and their genomes play pivotal roles in various biological processes, including programmed cell death, cytoplasmic male sterility, and responses to environmental stresses (Hanson and Bentolila, 2004; Van Aken and Van Breusegem, 2015). In addition, plant mitochondrial genomes are characterized by a low evolutionary rate, high structural plasticity, and predominantly maternal inheritance (Greiner et al., 2015; Gualberto and Newton, 2017). Therefore, elucidating the structure and organization of plant mitochondrial genomes is of great significance for understanding plant evolution, phylogeny, and molecular breeding (Arimura, 2018; Sloan et al., 2018).

The plant mitochondrial genome exhibits pronounced heterogeneity in various aspects, including genome size (ranging from several tens of kilobases to several megabases), structural configuration (circular, linear, branched, or multi-chromosomal forms), as well as in recombination frequency and repeat content. The assembly and analysis are further complicated by several factors: the presence of nuclear and plastid DNA inserts (known as NUMTs, nuclear mitochondrial DNA sequences, and NUPTs, nuclear plastid DNA sequences), the transfer of plastid sequences into the mitochondrial genome (termed MTPTs, mitochondrial plastid DNA sequences), and the widespread occurrence of allelic isoforms and substoichiometric configurations (He et al., 2023; Bi et al., 2024; Wang et al., 2024). These characteristics not only make the mitochondrial genome one of the most technically challenging organellar genomes to resolve but also provide a unique window into the understanding of genome evolution and intergenomic communication. Despite the rapid development of high-throughput sequencing technologies and novel assembly algorithms in recent years, achieving a complete and accurate reconstruction of plant mitochondrial genomes remains a formidable challenge (He et al., 2023; Bi et al., 2024; Tang et al., 2025). To address these challenges, several specialized software tools have been developed for mitochondrial genome assembly, such as GetOrganelle, GSAT, HIMT, PMAT, TIPPo, and PMAT2 (Jin et al., 2020; He et al., 2023; Bi et al., 2024; Han et al., 2025; Tang et al., 2025; Xian et al., 2025). Each of these tools possesses distinct advantages and limitations. In this paper, we review and compare these assembly approaches in light of recent studies, providing insights into future directions for plant mitochondrial genome research and development.

## 2 Modes of operation of different tools

### 2.1 GetOrganelle: a representative of short-read-based assemblers

GetOrganelle is a short-read-based assembly tool designed primarily for Illumina sequencing data. It employs a baiting-and-iterative-extension strategy to enrich organellar genome reads from whole-genome sequencing (WGS) datasets. The enriched reads are assembled using SPAdes to generate a FASTG file, which is then refined through coverage analysis and BLAST-based labeling to reduce redundancies. The pipeline outputs all resolvable circular configurations and provides a labeled assembly graph for semi-manual validation using Bandage. For circularization and path resolution, GetOrganelle adopts a DBG-based strategy: it estimates node multiplicity from coverage and graph connectivity, enumerates distinguishable paths, and accepts circularization only when the graph can be closed and multiplicity/coverage are consistent. Otherwise, it retains the GFA or linear contigs and recommends Bandage-assisted manual inspection. GetOrganelle has demonstrated stable and robust performance in chloroplast genome assembly and has also been widely applied to mitochondrial genome reconstruction (Jin et al., 2020). However, due to the abundance of repetitive sequences and structural variations within plant mitochondrial genomes, short-read-based assemblers often fail to bridge long repeat regions, making it difficult to obtain complete mitochondrial genome assemblies.

### 2.2 GSAT: an attempt at graph-based assembly approaches

GSAT employs a graph-structure-based strategy that initially constructs a draft organellar assembly graph from short-read data. Long reads are subsequently incorporated for alignment anchoring, error correction, and graph simplification, ultimately generating a mitochondrial master graph (MMG). Based on the spanning support of long reads, GSAT applies threshold filtering to remove low-frequency edges and eliminate low-abundance isoforms. In addition, the software visualizes the complete genomic architecture and the spectrum of allelic isoforms in a schematic manner, while systematically quantifying the interference effects of NUMTs and MTPTs (He et al., 2023). For circularization and path resolution, GSAT first builds an organelle graph from short reads and then calibrates/simplifies it with long reads. Major and minor edges are determined by long-read spanning frequency; low-frequency edges are filtered, and an MMG (mitochondrial main graph) is delivered. Circularization is accepted only when key junctions are long-read–spanned with coherent coverage. This strategy enables a panoramic structural characterization of the mitochondrial genome. However, its reliance on the short-read-based initial graph construction makes it susceptible to biases introduced by high-copy repeats and intergenomic homologous fragments. Consequently, read sorting and edge determination may be compromised, resulting in breakpoints, misjoins, or the omission of low-abundance configurations, ultimately leading to incomplete genome assemblies.

### 2.3 PMAT: an efficient tool for low-coverage HiFi data

PMAT is a recently developed assembly framework specifically designed for plant mitochondrial genomes. It enables genome reconstruction under ultra-low sequencing depth by first performing a whole-genome assembly, followed by the identification of mitochondrial contigs based on conserved protein-coding genes (PCGs), read coverage, and graph connectivity relationships. The pipeline subsequently generates a comprehensive panoramic assembly graph (GFA) (Bi et al., 2022, 2024). PMAT adopts a graph-driven strategy that allows the capture of multiple candidate configurations and isoforms. For circularization and path resolution, PMAT starts from high-confidence mitochondrial seeds and performs BFS expansion to construct a panoramic GFA. It then removes suspicious edges/nodes based on depth and link consistency, and exports all candidate conformations. Circularization is not enforced: closure is accepted only when the graph is closable and consistent with depth profiles and PCG support. It provides two operational modes-autoMito and graphBuild-to support both automated and semi-supervised (user-guided) workflows. Benchmarking across multiple plant species has demonstrated high accuracy and completeness of its assemblies. However, it should be noted that PMAT still requires a certain level of computational expertise for operation. Moreover, its scalability and robustness when dealing with extremely complex mitochondrial genomes remain to be systematically validated. Such complexity includes genomes characterized by high levels of rearrangement, repeat enrichment, or pronounced NUMT/NUPT interference, necessitating more comprehensive benchmarking studies.

### 2.4 TIPPo: a deep learning-driven approach

The core innovation of TIPPo lies in the integration of a sequence feature-based read classifier (TIARA) prior to the assembly stage. This classifier performs an initial screening of raw reads to identify potential organellar candidates, which are then refined through secondary filtering based on k-mer frequency analysis. This two-step classification effectively mitigates the interference caused by NUMTs and NUPTs, enabling organelle-specific genome assembly (Xian et al., 2025). Under conditions where nuclear insertions are abundant, TIPPo achieves substantial noise reduction through read sorting and allows for the simultaneous reconstruction of chloroplast and mitochondrial genomes. For circularization and path resolution, TIPPo follows a “read classification to assembly” workflow: it first applies TIARA plus k-mer-based denoising to suppress NUMTs/NUPTs, then performs organelle-specific assembly. Circularization and path choices are made only when long-read spanning and coverage coherence support a closed path; if ambiguity persists, the graph and candidate paths are retained. However, it should be noted that read classification remains subject to potential mis-sorting errors and species-specific biases. Moreover, the graphical representation of mitochondrial multi-conformational or isoform structures and the quantitative assessment of their relative abundances still rely on the integration and cross-validation with complementary assembly-graph tools.

### 2.5 HIMT: an integrated approach utilizing high-accuracy long reads

HIMT integrates HiFi sequencing data and automatically estimates genome coverage, employing a prefix-restricted k-mer strategy to reduce memory consumption. It also incorporates a graphical user interface (GUI), cross-platform compatibility, and interactive quality-control reports, thereby mitigating to some extent the interference from NUMTs (Tang et al., 2025). For circularization and path resolution, HIMT is oriented toward HiFi/ultra-long reads. It uses prefix-restricted k-mers to reduce memory and provides a GUI/QC workflow. Circularization is called only when HiFi reads span key junctions with coherent coverage; otherwise, the tool outputs graph or linear contigs with supporting evidence tracks, avoiding enforced circularization. The primary advantage of this approach lies in its effective exploitation of the low error rate and long-span capability of high-accuracy long reads, which substantially enhances both the continuity and accuracy of genome assembly. Nevertheless, the assembly process may still encounter limitations when dealing with mitochondrial genomes that have high repeat content, diverse isoforms, or multichromosomal configurations. These limitations typically manifest in read sorting, graph simplification, and conformation resolution. In such scenarios, validation through coverage-depth patterns, cross-read support, or genetic mapping is required to ensure assembly reliability.

### 2.6 PMAT2: a cross-lineage optimization of the PMAT framework

PMAT2 represents a systematic engineering upgrade built upon the original PMAT framework. The core modules have been reimplemented in C language to enhance computational efficiency, while lineage-specific optimizations have been introduced to support diverse taxa-including animals, fungi, plants, and plastomes. The exhaustive path-searching algorithm used in PMAT has been replaced by a BFS-seed strategy combined with hash indexing, substantially improving performance and scalability. PMAT2 further integrates an automated assembly evaluation module, which assesses assembly quality by jointly considering gene completeness, genome scale, and rearrangement signals. For circularization and path resolution, PMAT2 builds on PMAT by incorporating BFSseed/bfsMap with hashing to accelerate path search and scoring, applying depth thresholds, and down-weighting MTPT/NUPT segments to reduce mislinks. It reports closed or multi-circular solutions only when long-read spanning and coverage coherence are satisfied; otherwise, it retains the panoramic GFA with quantified support. Empirical tests have demonstrated that PMAT2 can achieve high-quality, graph-based assemblies across multiple taxonomic groups even under ultra-low HiFi sequencing coverage, offering both broad applicability and strong usability under low-depth conditions.

To provide a more intuitive comparison of the advantages and limitations of different tools, their key features are summarized below (**Table 1**).

**Table 1:** Comparison of commonly used software for plant mitochondrial genome assembly.

Software	Data type	Major advantages	Major limitations	Recommended application scenarios
GetOrganelle	Illumina short reads	Mature algorithm; easy to operate	Unable to span long repeats, leading to incomplete assemblies	Suitable for chloroplast genomes; applicable to simple mitochondrial genomes only
GSAT	Illumina + long reads	Capable of revealing multiple genome conformations	Dependent on short-read-based initial graph, may result in incomplete assemblies	Suitable for studies on mitochondrial genome structural complexity
PMAT	Low-coverage HiFi reads	Highly efficient; performs well even at 1× coverage; supports panoramic structural representation	Requires computational expertise; needs further validation for highly complex genomes	Broadly applicable across diverse plant species
TIPPo	HiFi reads	Effectively filters NUMTs/NUPTs; user-friendly interface	May be less stable than PMAT for certain genomes	Recommended as a general-purpose organelle genome assembler
HIMT	HiFi/ONT long reads	High accuracy; reduces NUMT interference	Limited performance on highly repetitive genomes	Suitable for mitochondrial assembly from high-quality sequencing data
PMAT2	Low-coverage HiFi reads	Addresses PMAT’s limitations for complex genomes; improved cross-lineage performance	MTPT/NUMT interference mitigated but not completely eliminated	Broadly applicable across multiple taxonomic groups

## 3 Discussion

Overall, the paradigm of plant mitochondrial genome assembly has shifted from a reliance on short-read data to a long-read-dominated framework, incorporating graph-based algorithms and data-driven read classification. This integrated approach enhances assembly continuity and interpretability. Organelle assembly still faces several challenges: (i) graph ambiguity and unresolved path choices caused by long repeats, frequent rearrangements, and heteroplasmy; (ii) read misclassification under NUMTs/MTPTs contamination, propagating to erroneous links; (iii) underestimation and biased quantification of sub-stoichiometric isoforms under low or uneven coverage; (iv) the lack of standardized, graph-level deliverables and benchmarks; and (v) insufficient mult. Therefore, several critical issues need to be addressed.

1. Standardization of graph-based deliverables:

In publication and data sharing, it is recommended that, in addition to the linearized sequences, the assembly graph (GFA) and corresponding long-read mapping evidence (coverage depth and path-support frequency) be provided. The edge sets and threshold criteria distinguishing primary from secondary or rare structures should also be clearly defined. The MMG model in GSAT and the panoramic graph-based paradigms in PMAT/PMAT2 could serve as generalized templates for standardized reporting.

2. Quantification and reporting of isoforms:

Leveraging HiFi or ultra-long read data, isoform quantification can be achieved by path counting and depth normalization on assembly graphs, allowing the reporting of support frequency intervals for each connection or conformation. When necessary, read-backed phasing or haplotyping strategies should be employed to avoid oversimplifying complex multiconformational systems into a single circular model.

3. Integration of read classification and graph disambiguation:

By adopting TIPPo’s read classification to mitigate NUMT/NUPT interference, and subsequently coupling it with graph-based assembly frameworks such as PMAT2 or GSAT, the processes of connection verification and conformation quantification can be integrated. This composite workflow, which integrates “read sorting and denoising + graph-based validation and quantification”, enhances the accuracy, completeness, and interpretability of assemblies. It is particularly effective for resolving complex mitochondrial genomes.

4. Annotation and benchmarking standards:

In mitochondrial genome annotation, it is advisable to annotate not only protein-coding genes (PCGs), tRNAs, and rRNAs, but also RNA editing sites and potential MTPT/NUMT fragments, marking their graph positions and adjacency relationships. Furthermore, establishing an open benchmarking dataset covering multiconformational, repeat-spanning, and insertion-containing cases would facilitate community-level evaluations at the graph scale.

5. Criteria for assessing assembly completeness.

Assess completeness across three tiers: sequence (Merqury QV/k-mer recovery, BUSCO with lineage/version, mapping breadth and median depth); structural/graph (GFA closure only with long-read spanning of repeats/junctions; document bubble/repeat resolution; report alternative isoforms with edge/path support and frequencies); and annotation/contamination (PCG/tRNA/rRNA coverage; systematic screening/down-weighting of NUMTs/MTPTs). Also report tool versions/parameters/thresholds and provide FASTA + GFA with long-read alignment evidence.

6. Cost and throughput considerations:

The strong usability of PMAT/PMAT2 under low sequencing depth substantially lowers the entry barrier for mitochondrial genome assembly. Combined with the GUI and containerized one-click workflows of HIMT, large-scale batch assembly of “master graphs + alternative conformations’’ across populations or breeding materials becomes feasible, providing a valuable data foundation for studies on cytoplasmic male sterility (CMS) and nuclear-cytoplasmic interactions.

7. Integration with complementary evidence:

For critical connections or conformations, integration of multi-source validation evidence such as Bionano maps, Hi-C data, ultra-long ONT reads, or PCR-Sanger verification is recommended. These evidence tracks should be retained within the assembly graph, embedding reproducibility and verifiability directly into the data product.

Future work should focus on several key areas. First, coupling deep learning-based read classification with graph algorithms could enable joint optimization of read-level features and graph-level constraints. Second, the development of highly automated, interactive visualization interfaces is needed to make these tools accessible to non-specialists. Finally, the construction of unified benchmarking datasets—encompassing multi-conformational, repeat-spanning, and insertion-rich cases—is essential for establishing community-wide methodological and reporting standards.

In summary, the field of plant mitochondrial genome assembly is being propelled by the rapid iteration of software tools. Continuous innovations-from GetOrganelle to PMAT2-have significantly advanced the field. With the co-evolution of sequencing technologies and algorithmic frameworks, the panoramic reconstruction of plant mitochondrial genomes is expected to become routine. This capability will profoundly improve our understanding of organelle evolution and genome interactions and provide high-quality, reusable resources and methodological foundations for plant breeding and applied research. Moreover, these more complete and accurately resolved mitochondrial assemblies enable concrete biological applications by improving studies of plant adaptive evolution, clarifying mitochondrial contributions to energy metabolism, and facilitating the identification of cytoplasmic variants relevant to breeding and stress resilience.
